# Long-term clinical and cost-effectiveness of collaborative care (versus usual care) for people with mental–physical multimorbidity: cluster-randomised trial

**DOI:** 10.1192/bjp.2018.70

**Published:** 2018-08

**Authors:** Elizabeth M. Camacho, Linda M. Davies, Mark Hann, Nicola Small, Peter Bower, Carolyn Chew-Graham, Clare Baguely, Linda Gask, Chris M. Dickens, Karina Lovell, Waquas Waheed, Chris J. Gibbons, Peter Coventry

**Affiliations:** 1 Division of Population Health, Health Services Research, and Primary Care, The University of Manchester, UK; 2 NIHR School for Primary Care Research, The University of Manchester, Manchester Academic Health Science Centre, UK; 3 Primary Care & Health Sciences, University of Keele, UK, Division of Population Health, Health Services Research, and Primary Care, The University of Manchester, UK and NIHR Collaboration for Leadership in Applied Health Research and Care West Midlands, UK; 4 NHS Health Education North West, Manchester, UK; 5 Division of Population Health, Health Services Research, and Primary Care, The University of Manchester, UK; 6 Mental Health Research Group, University of Exeter, UK; 7 Division of Nursing, Midwifery and Social Work, The University of Manchester, Manchester Academic Health Science Centre, UK; 8 Division of Population Health, Health Services Research, and Primary Care, The University of Manchester, UK; 9 The Psychometrics Centre, University of Cambridge, UK and Division of Population Health, Health Services Research, and Primary Care, The University of Manchester, UK; 10 Department of Health Sciences, University of York, UK and Centre for Reviews and Dissemination, University of York, UK.

## Abstract

**Background:**

Collaborative care can support the treatment of depression in people with long-term conditions, but long-term benefits and costs are unknown.

**Aims:**

To explore the long-term (24-month) effectiveness and cost-effectiveness of collaborative care in people with mental-physical multimorbidity.

**Method:**

A cluster randomised trial compared collaborative care (integrated physical and mental healthcare) with usual care for depression alongside diabetes and/or coronary heart disease. Depression symptoms were measured by the symptom checklist-depression scale (SCL-D13). The economic evaluation was from the perspective of the English National Health Service.

**Results:**

191 participants were allocated to collaborative care and 196 to usual care. At 24 months, the mean SCL-D13 score was 0.27 (95% CI, −0.48 to −0.06) lower in the collaborative care group alongside a gain of 0.14 (95% CI, 0.06-0.21) quality-adjusted life-years (QALYs). The cost per QALY gained was £13 069.

**Conclusions:**

In the long term, collaborative care reduces depression and is potentially cost-effective at internationally accepted willingness-to-pay thresholds.

**Declaration of interest:**

None.

## Relevance statement

Collaborative care integrates physical and mental healthcare. It is more effective than usual care for managing depression over the long term in people with coexisting mental and physical health conditions (mental–physical multimorbidity). This is the first long-term evaluation of collaborative care for managing mental–physical multimorbidity in a UK setting. Additionally, collaborative care is good value for money over the long term and has the potential to deliver important health gains at levels generally considered to be cost-effective.

## Introduction

Multimorbidity refers to the presence of two or more long-term conditions, which can include combinations of physical and mental symptoms. Multimorbidity is a critical burden on health systems, associated with increased mortality, reduced quality of life and increased use of unscheduled care.^1^^,^^2^ The greatest reductions in quality of life are experienced by those with mental–physical multimorbidity^3^ which is associated with 45–65% higher costs of care for long-term conditions.^4^ Collaborative care is a model of care for people with co-occurring mental and physical health conditions that recognises the interplay between the two. The most widely accepted current definition of collaborative care includes four key criteria: a multi-professional approach to patient care, a structured management plan, scheduled patient follow-ups and enhanced inter-professional communication.^5^ A key element is the appointment of a care manager who acts as a conduit between patients and healthcare professionals, and works with the patient to promote better patient self-care.^6^ There is some evidence that compared with usual care, collaborative care is more effective for treating depression and anxiety over the short to medium term, with or without multimorbid long-term conditions,^7^ but effectiveness beyond 12 months remains uncertain. The UK National Institute for Health and Care Excellence (NICE) concluded that there is currently an absence of clinical or cost-effectiveness evidence for collaborative care in multimorbidity.^8^ In the context of interventions for long-term health conditions it is especially important to evaluate long-term clinical effectiveness and cost-effectiveness. This article reports the long-term (24-month) clinical and cost-effectiveness of collaborative care for people with depression, in the context of high levels of multimorbidity, as part of the Collaborative Interventions for Circulation and Depression (COINCIDE) trial.

## Method

### Trial design and participants

The COINCIDE trial evaluated the clinical effectiveness of collaborative care over a short-term period (4 months); collaborative care was associated with a significantly greater improvement in depression symptoms compared with usual care (effect size: 0.30).^9^ The study protocol (trial registration number: ISRCTN80309252) has been published previously.^6^^,^^10^

The evaluation was a two-arm, cluster randomised, controlled trial in the north-west of England. Participating general practices held electronic registers of patients with diabetes and/or coronary heart disease and were assigned a unique identifier. A total of 459 general practices across the north-west of England were invited to participate and 39 were allocated in a phased approach across the region (9 in Merseyside, 19 in Greater Manchester and 8 in East Lancashire); three practices withdrew before participants were recruited. Designated staff based remotely at the Clinical Trials Unit of the Christie National Health Service (NHS) Foundation Trust (Manchester, UK) provided a central randomisation service. A computerised random number generator was used. The first six practices recruited were allocated at a ratio of 1:1 at random, to either the collaborative care or usual care arm. Subsequent practices were then allocated by minimisation (with a probability weighting of 0.8),^11^ based on the Index of Multiple Deprivation and practice list size. An email confirmation of allocation details was sent to the trial manager. Research staff were blind to the allocation of enrolled participants. Details of sample size calculations are reported in full elsewhere;^9^ the trial had 79% power to detect an effect size of 0.4.

Staff from the National Institute for Health's Mental Health Research Network searched clinical databases at participating practices for patients with a record of diabetes and/or coronary heart disease. Patients who met these eligibility criteria were sent a postal invitation followed by a reminder letter 3 weeks later; non-responders to the reminder postal invitation were telephoned. Patients were screened for depressive symptoms (≥10 on the nine-item Patient Health Questionnaire; PHQ-9)^12^ by the research team over the telephone twice over 2 weeks. Patients who met these criteria for at least 2 weeks were eligible to participate. We excluded patients with psychosis, patients with type I or type II bipolar disorder, those who were currently experiencing suicidal thoughts, those in receipt of services for substance misuse and those receiving psychological therapy for depression from a mental health service. Full details of the trial design are reported in detail in the published protocol.^6^^,^^10^

### Ethical approval

The National Research Ethics Service Committee North West – Preston (approval number NRES/11/NW/0742) granted ethical approval, and research governance approvals were granted by participating primary care trusts. Informed consent was obtained in writing from all participants before data collection.

### Interventions

Participants attending general practitioner (GP) practices allocated to the collaborative care arm received up to eight face-to-face sessions of brief psychological therapy delivered by a case manager over 3 months.^6^^,^^10^ Case managers were ‘psychological well-being practitioners’ (PWPs) employed by Improving Access to Psychological Therapy (IAPT) services, given specific training in delivering the COINCIDE collaborative care model. The first session was expected to last for 45 min, during which the PWP identified links between participants' mood and management of their long-term conditions with the aim of formulating a problem statement. Subsequent treatment sessions were scheduled to last for 30–40 min and participants could choose to engage with behavioural activation, graded exposure, cognitive restructuring and/or lifestyle changes. A 10 min collaborative meeting (by telephone or in person) between the participant, PWP and a practice nurse from the participant's general practice was scheduled to take place during treatment session two and eight, to facilitate the integration of care. These collaborative meetings focused on ensuring that psychological treatments did not complicate current management, reviewing patients' progress, reviewing relevant physical and mental health outcomes and planning future care.^9^ The final session also included education about relapse prevention strategies. PWPs were expected to liaise with the practice nurse and participants' GPs about medication and update on participant progress. Participants attending general practices allocated to the control arm received treatment as usual based on the NICE stepped care model, provided by the participants' GPs.^13^ This could include treatment of depression with medication and/or onward referral to psychological therapy. If participants in the usual care arm were referred to psychological therapy provided for by Improving Access to Psychological Therapy services, they did not receive such care from COINCIDE-trained PWPs.

### Outcomes

The primary outcome was self-reported depression severity on the 13 depression items of the Symptom Check List-90 scale (SCL-D13; range: 0–4) 24 months after randomisation.^14^ Data on healthcare utilisation were collected with a bespoke patient questionnaire. Health status was measured with the EuroQol 5D-5L (EQ-5D-5L).^15^ All baseline measures were collected face to face by research staff blind to allocation; self-reported outcome measures at 24 months were collected by postal questionnaires. Participants who did not return the 24-month questionnaire were contacted by telephone and given the opportunity to complete the primary outcome over the telephone with a researcher blind to allocation.

For economic analyses, we used the UK NHS and personal social services perspective in line with NICE guidance for economic evaluations of healthcare interventions.^16^ The time horizon for the economic evaluation was 24 months. Data on resource utilisation were collected at 4 months (covering the period between baseline and 4 months) and 24 months (covering the period between 4 and 24 months). Therefore, it was not possible to distinguish which visits occurred in the period between 4 and 12 months and which occurred between 12 and 24 months. For this reason, costs associated with healthcare utilisation beyond 12 months were not discounted. For comparability, outcomes were also not discounted.

Use of healthcare services was collected by asking participants to report their total number of visits to different healthcare professionals in these categories: in-patient admission, out-patient, day patient (non-overnight hospital admission), accident and emergency, and primary/ community care (e.g. GP, nurse, social worker). Direct costs were also estimated for the intervention. Data on the resources used to provide the intervention only were collected from activity logs completed by PWPs and practice nurses who delivered care to participants in the collaborative care arm. The costs of training PWPs were also included in the primary economic analysis. We based PWP costs on NHS Agenda for Change salary band four (current salary range: £19 217–22 458; US$24 271–28 364; €21 793–25 469) and included employer National Insurance and pension contributions plus capital, administrative and managerial costs. We calculated cost per hour using standard working time assumptions,^17^ weighted to account for time spent on non-patient-facing activities. For each type of resource, the cost was estimated as the quantity of that resource or service used multiplied by nationally applicable unit costs.^17^^,^^18^ All direct costs are reported in British pounds (£) and inflated to 2015–2016 prices by the Hospital and Community Health Services Index.^17^

The primary measure of cost-effectiveness was cost per quality-adjusted life-year (QALY).^16^ Utility values were derived at each time point from the EQ-5D-5L and associated utility tariffs for the UK, generated from a series of time trade-off and discrete choice experiments.^19^ The EQ-5D-3L crosswalk method^20^ was used to estimate utility values as a sensitivity analysis. A mean utility value was calculated for each set of two time points (baseline to 4 months; 4–24 months). The mean utility value and length of the respective time period (e.g. a utility value of 1 is equivalent to 0.5 QALYs if accrued over 6 months, or 1 QALY if accrued over 12 months) was used to generate two QALY values (one for each time period), which were combined to estimate the total QALYs during the whole follow-up period.

### Statistical analysis

We used an intention-to-treat approach for all clinical and cost-effectiveness analyses as per the original data analysis plan shared with the Data Monitoring and Ethics Committee. All analyses were conducted with Stata (Release 13). The trial originally had 80% power (two-sided α = 0.05) to detect a difference between groups on the primary outcome at follow-up, equivalent to a standardised effect size of 0.4, for which we required 15 practices per arm and 15 patients per practice (*n* = 450), allowing for 20% attrition and an intra-practice correlation of 0.06. Average recruitment in the first 11 practices was <15 patients per practice. We therefore increased the total number of clusters from 30 to 36, with a target of 10 patients per practice, giving 79% power to detect an effect of 0.4 under the same assumptions. The revised target sample was therefore 360 patients.^10^

To compare the change in depression scores between participants randomised to collaborative care or usual care, multiple linear regression with robust standard errors was used; this accounted for the clustering of patients within practices. The following baseline characteristics were controlled for: depression score, age, gender, area deprivation (based on residential postcode), level of limitation of daily activities at baseline, use of antidepressants or antianxiety drugs (currently, previously or never), GP practice list size and GP practice area deprivation (the latter two as ‘design factors’). Multiple imputation was used to estimate missing scale scores and other data values at both baseline and follow-up. Thirty imputed data-sets were generated with chained equations, including covariates as listed above. As part of the imputation model, missing SCL-D13 values were restricted to between 0 and 4, in line with the possible questionnaire responses. To assess sensitivity of the results to multiple imputation, we conducted secondary analyses on complete cases, first with the same covariates as the main analysis and second with only baseline depression score included as a covariate. A further *post hoc* sensitivity analysis was conducted without restricting the range of imputed SCL-D13 values.

### Economic analysis

The base–case (primary) economic analysis calculated incremental cost-effectiveness ratios (ICERs); accordingly, no parametric statistical tests of differences in mean costs or outcomes were conducted. Net QALYs (collaborative care versus usual care) were estimated by a linear regression model, and net costs were estimated by a generalised linear regression model with a log link and gamma family to allow for the non-normal distribution of costs. Regression models were adjusted for baseline variables identified through stepwise regression, using the following measures: World Health Organization Quality of Life Instrument,^21^ Generalised Anxiety Disorder seven-item Scale,^22^ PHQ-9, Self-Efficacy Questionnaire,^23^ Health Education Impact Questionnaire,^24^ Sheehan Disability Scale,^25^ employment status and mobility (EQ-5D-5L). Robust standard errors were used to account for the clustering of patients within practices. The estimates of incremental costs and outcomes from the regression were bootstrapped to simulate either 2000 or 10 000 pairs of net cost and net outcomes for a cost-effectiveness acceptability analysis, as recommended by NICE for health technology appraisals.^16^

Missing data on costs and EQ-5D-5L utility scores were imputed five times with a chained-equation procedure. Costs were imputed by category and utility by individual EQ-5D-5L domain rather than as totals, so that all available data were used to inform the imputed values. The pattern of available cost and utility data across the different assessments are summarised in supplementary Table 1, available at https://doi.org/10.1192/bjp.2018.70. For the 24-month assessment, 69% of the original sample had a cost recorded for at least one category of healthcare and 69% had responded to at least one item on the EQ-5D-5L. All available (complete and partial) cost and outcome data for a particular participant was used to impute missing data. The number of imputed data-sets was chosen for pragmatic reasons: it was felt that this represented a balance between robustness and the computational burden of conducting the bootstrapping procedure on imputed data.

Sensitivity analyses assessed the effect of design and analysis choices on the cost-effectiveness of collaborative care. These were an alternative method for utility/QALY estimation (crosswalk approach),^20^ a complete case analysis, an alternative measure of health benefit (proportion of participants showing a ‘response’ – 40% improvement from baseline in SCL-D13 score),^26^ excluding the cost of training the PWPs and number of bootstrap simulations.

## Results

19 practices were randomised to collaborative care and 20 to usual care. We identified 387 patients with depression and heart disease and/or diabetes and invited them to a baseline assessment. Follow-up data on 350 (90% of original sample) participants were collected at 4 months between 18 November 2012 and 4 October 2013, and follow-up data on 272 participants (71% of original sample) were collected at 24 months between 18 May 2014 and 4 June 2015 (see supplementary material for the Consolidated Standards of Reporting Trials (CONSORT) diagram). Characteristics of participating practices and participants are reported in Table 1. The majority (63%) of participants at baseline met criteria for moderately severe or severe depression, and 75% of participants met criteria for anxiety. Participants reported a mean of 6.2 (s.d. = 3.2) long-term conditions in addition to either diabetes or coronary heart disease; 15% of participants had a diagnosis of both diabetes and coronary heart disease. The mean age was 58.5 years and 38% of participants were female. Around half (54%; *n* = 211) of participants lived in areas ranked as highly deprived (index of multiple deprivation score ≥30). A total of 25% of participants were in paid employment. Half of participants were prescribed antidepressant or antianxiety medication at baseline. Full details about the delivery of the intervention have been previously reported.^9^

**Table 1:** Baseline characteristics of COINCIDE trial practices and participants

	Collaborative care (*n* = 191)	Usual care (*n* = 196)
	*n* (%)	*n* (%)
Practice area		
Affluent	49 (26)	43 (22)
Moderately deprived	80 (42)	90 (46)
Heavily deprived	62 (33)	63 (32)
Practice size		
Small (<4500)	60 (31)	49 (25)
Medium (4500 to 7500)	61 (32)	41 (21)
Large (>7500)	70 (37)	106 (54)
Participant characteristics		
Age in years^a^	57.9 (12.0)	59.2 (11.4)
Gender female	78 (41)	69 (35)
Ethnicity White	162 (85)	167 (85)
QOF register diabetes	106 (56)	101 (51)
QOF register CHD	56 (29)	66 (34)
QOF register diabetes and CHD	29 (15)	29 (15)
	Mean (s.d.)	Mean (s.d.)
Deprivation IMD score (low score indicates high deprivation)	36.6 (21.3)	34.4 (18.5)
Number of long-term physical conditions^b^	6.0 (3.2)	6.5 (3.1)
PHQ-9 total depression score (0–27)	16.4 (4.2)	16.5 (4.1)
GAD-7 total anxiety score (0–21)	12.3 (5.1)	11.9 (5.3)

COINCIDE, Collaborative Interventions for Circulation and Depression; QOF, quality outcomes framework; CHD, coronary heart disease; IMD, Index of Multiple Deprivation; PHQ-9, Patient Health Questionnaire 9; GAD-7, Generalised Anxiety Disorder seven-item Scale.

a. Mean (s.d.).

b. Excluding diabetes and CHD.

For the primary outcome measure, depression scores were available at 24 months for 62% of participants allocated to the collaborative care arm and 74% allocated to usual care. The primary measure of health benefit for the economic evaluation was completed by 61% of collaborative care participants and 68% of usual care participants at 24 months. Across the categories of healthcare utilisation, 70–83% of participants had complete data at 4 months and 64–68% at 24 months. However, when combined into a total cost, 34% of the collaborative care group and 40% of the usual care had data for all of the categories at both time points. An additional summary of available cost and utility data is reported in supplementary Table 2.

**Fig. 1:**
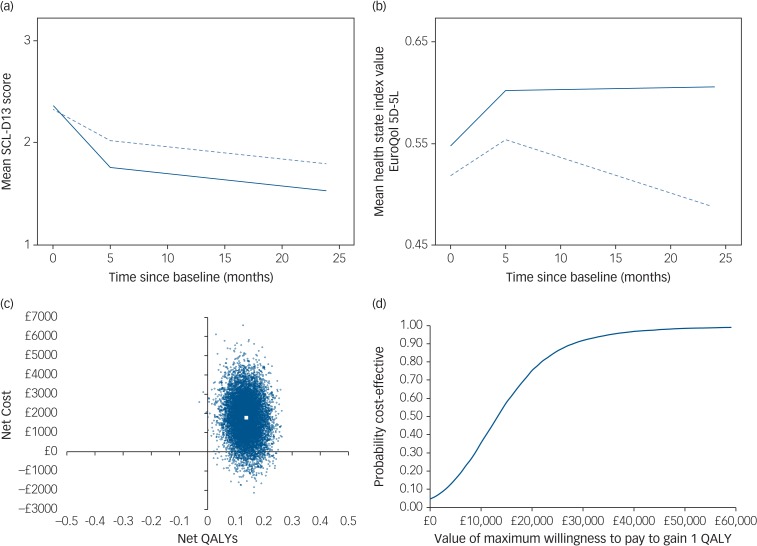
Summary of clinical effectiveness and cost-effectiveness results. (a) Mean Symptom Checklist-13 Depression Scale (SCL-D13) scores during follow-up by treatment group, unadjusted values (solid line represents collaborative care; dashed line represents usual care). (b) Mean health state index (EuroQol 5D-5L) scores during follow-up by treatment group, unadjusted values (solid line represents collaborative care; dashed line represents usual care). (c) Cost-effectiveness plane (primary analysis): distribution of 10 000 bootstrapped simulations of net cost and net quality-adjusted life-year (QALY) pairs (large white square indicates point estimate for incremental cost-effectiveness ratio). (d) Cost-effectiveness acceptability curve (primary analysis).

The unadjusted difference in mean SCL-D13 scores between baseline and first follow-up showed an improvement in both groups (4-month improvement: collaborative care 0.61; usual care 0.31) (Table 2 and Figure 1a). When compared with baseline values, 24-month depression scores were again lower in both groups, with the greater improvement maintained in the collaborative care arm (24-month improvement: collaborative care 0.84; usual care 0.55).

**Table 2:** Mean depression scores (SCL-D13) at all time points and change in depression scores between baseline and 24 months

	Collaborative care		Usual care	
	*n*	Mean (s.d.)	*n*	Mean (s.d.)
Baseline	191	2.364 (0.696)	196	2.330 (0.822)
4 months	170	1.756 (0.938)	180	2.020 (0.935)
24 months	119	1.527 (0.945)	145	1.785 (1.034)
Attrition rate (baseline to 24 months)	37.7%		26.0%	
	Adjusted^a^ difference in means		Effect size	
	(95% CI)		(95% CI)	
Primary analysis				
Imputed data (*n* = 387)	−0.269 (−0.476 to −0.061)		−0.353 (−0.624 to −0.047)	
Sensitivity analysis				
Complete cases (*n* = 245)	−0.264 (−0.450 to −0.078)		−0.346 (−0.454 to −0.102)	
Complete cases (adjusted for baseline SCL-D13 score only) (*n* = 264)	−0.260 (−0.416 to −0.104)		−0.341 (−0.546 to −0.136)	
Imputed data, regression model not constrained to possible value range (0–4) for mean SCL-D13 (*n* = 387)	−0.260 (−0.416 to −0.104)		−0.341 (−0.546 to −0.136)	

SCL-D13, Symptom Checklist-13 Depression Scale.

a. Adjusted for all following covariates unless otherwise indicated: age, gender, socioeconomic deprivation, limitation of daily activities owing to comorbidities, use of antidepressants or antianxiety drugs and general practitioner practice characteristics.

After adjustment for covariates, the mean SCL-D13 score at 24-month follow-up was 0.27 points lower (95% CI, −0.48 to −0.06; *P* = 0.014) in participants who received collaborative care compared with those who received usual care. This difference is equal to a standardised mean difference of −0.35 (95% CI, −0.62 to −0.05), using the baseline pooled s.d. for SCL-D13. Collaborative care was also found to be significantly more effective than usual care at 24 months in all sensitivity analyses, although there was very little difference in the estimated coefficients between models (Table 2).

### Cost-effectiveness

The mean cost of the collaborative care intervention was £321, including a training cost of £130 per participant (total training costs divided by the total number of participants randomised to collaborative care irrespective of number of PWP sessions attended). For participants with complete cost data at all time points, the mean cost across all categories of healthcare was higher among those randomised to collaborative care; the cost of healthcare resources used was higher across all categories except day patient hospital visits and emergency department visits (see supplementary Table 3).

Unadjusted mean health state index scores show that the usual care group worsened overall between baseline and 24 months (Figure 1b; also see supplementary Table 3). The collaborative care group improved between baseline and first follow-up, which was sustained at the 24-month follow-up.

Regression analysis on multiple imputed data-sets also showed a higher net cost associated with collaborative care (compared with usual care); £1777 (95% CI, −£320 to £3875) over 24 months, although this difference is not statistically significant. Participants receiving collaborative care accrued significantly more QALYs over 24 months than those receiving usual care (0.136; 95% CI, 0.061 to 0.212). The bootstrapped estimates of net costs and QALYs are shown on a cost-effectiveness plane in Figure 1c. The simulations are predominantly located in the upper-right quadrant of the plane, indicating a net cost associated with collaborative care alongside a net health benefit (QALY gain). The points on the plane show more vertical than horizontal spread, illustrating that there is greater uncertainty around the estimated net cost than the estimated net benefit.

The cost per additional QALY gained from collaborative care (compared with usual care) is £13 069. The probability of collaborative care being cost-effective is 0.75 if decision makers are willing to pay £20 000 to gain one QALY. If decision makers are willing to pay £30 000 to gain one QALY, the probability that collaborative care is more cost-effective than usual care is 0.92. This is shown in the cost-effectiveness acceptability curve in Figure 1d.

There was little difference in the 95% confidence intervals generated from non-parametric bootstrapping 2000 or 10 000 pairs of net costs and benefits, and no effect on the cost-effectiveness recommendation (Table 3). When the crosswalk method was used for estimating utility values from the EQ-5D-5L, the net QALYs were slightly lower than for the time trade-off approach (0.118 *v.* 0.136), resulting in a slightly higher ICER (£15 063/QALY *v.* £13 069/QALY). When only participants with complete cost and QALY data were included in the analysis, net costs were higher and net QALYs lower than the primary (base–case) analysis. This resulted in an ICER of £38 032 per QALY at which collaborative care would be unlikely to be more cost-effective than usual care (probability 0.42 at a willingness-to-pay threshold of £30 000). When the measure of health benefit was the number of people showing a clinical response in terms of depression symptoms (40% reduction in total SCL-D13 score^26^ between baseline and 24 months), there were 50 more ‘responders’ in the collaborative care group. Alongside the net cost of £1777, the cost per each additional responder was estimated to be £36. There is no guidance on how much decision makers are willing to pay for a treatment response, therefore it is not meaningful to calculate a probability of cost-effectiveness in terms of this outcome measure.

**Table 3:** Net costs and QALYs, ICER and probability collaborative care is cost-effective, using primary and sensitivity analyses, adjusted for baseline covariates, bootstrapped and imputed data (unless otherwise stated)

	Net cost (95% CI)	Net QALY (95% CI)	ICER (£/QALY)	Probability collaborative care is cost-effective versus usual care if WTPT =		
				£15 000/ QALY	£20 000/ QALY	£30 000/ QALY
Primary analysis						
Multiple imputation (*n* = 387), 10 000 bootstrap simulations	1777 (−320 to 3875)	0.136 (0.061–0.212)	£13 069/QALY	0.58	0.75	0.92
Multiple imputation (*n* = 387), 2000 bootstrap simulations	1777 (−313 to 3867)	0.136 (0.061–0.212)	£13 069/QALY	0.57	0.75	0.92
Sensitivity analyses (2000 bootstrap simulations)						
QALYs estimated with crosswalk methodology^20^	1777 (−313 to 3867)	0.118 (0.001–0.235)	£15 063/QALY	0.49	0.63	0.78
No imputation (complete cases, *n* = 130)	3347 (−1119 to 7813)	0.088 (−0.060 to 0.237)	£38 032/QALY	0.21	0.28	0.42
Excluding PWP training costs	1632 (−457 to 3722)	0.136 (0.061–0.212)	£12 002/QALY	0.62	0.78	0.93
		Number of responders (95% CI)	Cost per additional person responding to treatment			
Health benefit: ‘response’ on SCL-13 (40% improvement from baseline)^21^	1777 (−320 to 3875)	Usual care: 35 (25–45), collaborative care: 85 (72–98), difference: 50	£36^a^	–	–	–

Covariates costs: baseline mobility (EuroQol-5D), general practitioner practice (cluster). Covariates QALYs: baseline scores for World Health Organization Quality of Life Instrument, Generalised Anxiety Disorder seven-item Scale, Patient Health Questionnaire 9, Self-Efficacy Questionnaire, Health Education Impact Questionnaire, Sheehan Disability Scale, employment status and general practitioner practice (cluster).

QALY, quality-adjusted life-year; ICER, incremental cost-effectiveness ratio; WTPT, willingness-to-pay threshold; PWP, psychological well-being practitioner; SCL-D13, Symptom Checklist-13 Depression Scale.

a. Net cost (£1777) divided by the number of additional responders (50).

## Discussion

Collaborative care for depression in the context of multimorbidity is clinically and cost-effective over the long term. Not only were the treatment effects of collaborative care maintained over 24 months, but they marginally exceeded those reported at 4 months (standardised mean difference 0.35 *v.* 0.30). Collaborative care was also associated with superior QALY gains compared with usual care (i.e. when both mental and physical domains of health are considered) in this context.

This is the first long-term evaluation of collaborative care for managing mental–physical multimorbidity in a UK primary care setting. The net QALY gain observed in the COINCIDE trial at 24 months was greater than that observed in a previous economic evaluation of collaborative care in UK primary care.^27^ We previously reported results of an economic model estimating the long-term cost-effectiveness of collaborative care, based on data observed at 4 months in the COINCIDE trial.^28^ The net QALY gain observed here from 24-month trial data (0.14) was similar to the modelled scenario, which assumed that the benefit of collaborative care accrued at 4 months would be maintained at the same level over 24 months (net QALY gain 0.15). Our findings here support this assumption; the mean utility values were similar at 4 and 24 months in the collaborative care group, although the ICER estimated from the model was somewhat lower than for the 24-month trial data (£3468/QALY (model) *v.* £13 069/QALY (trial)).

An important difference between the economic model and observed data is the estimated net cost. Net costs were notably higher using data observed within-trial, meaning that the additional healthcare resources used by participants randomised to collaborative care was greater than expected. Participants in the collaborative care arm used almost £1800 more of healthcare resources over 24 months (crudely, £900 over 12 months) than those receiving usual care. By comparison, in the Clinical and Cost Effectiveness of Collaborative Care for Depression in UK Primary Care Trial (CADET) the cost of healthcare resources over 12 months were almost identical between usual and collaborative care treatment groups.^27^ The unexpectedly high resource use might partly be explained by participants in the collaborative care arm reporting improved navigation of health services and more engagement with health-directed behaviours, as evidenced by higher ratings on the Health Education Impact Questionnaire, compared with usual care.^9^ This may have led to increased but more appropriate use of healthcare resources and thereby improved levels of both physical and mental functioning.

### Strengths and limitations

The COINCIDE trial was a large, pragmatic trial conducted across a wide geographical area in the north-west of England that included a population with high levels of multimorbidity, disability and deprivation. This makes it particularly relevant to the management of mental–physical multimorbidity, which is more prevalent in younger adults from deprived regions.^29^ Cluster randomisation and analytic approaches for imputing data and adjusting for baseline characteristics offered greater opportunities to minimise bias and confounding. Additionally, the 24-month follow-up period is the longest time horizon used in an integrated economic and effectiveness evaluation of collaborative care outside the USA.

Our economic analysis conforms to the high standards of analysis and reporting expected of cost-effectiveness analyses of health interventions (see supplementary material for the Consolidated Health Economic Evaluation Reporting Standards checklist). However, data was not captured at baseline regarding use of health services before the intervention so it is not possible to determine or adjust for any underlying differences between the treatment groups.

Although multiple imputation of missing data reduced the potential for bias associated with missing data, the robustness of any imputation method declines as the level of missing data increases, reducing the validity and reliability of the analyses. Data from baseline and 24 months only were included in the clinical effectiveness analysis, and there was little difference in the change in depression score or effect size between the complete case analysis and the analysis after multiple imputation. The attrition rate was higher among participants in the collaborative care group (37.7%) compared with usual care (26.0%). We have explored key characteristics between participants with and without complete data (results not shown) and found only that in the usual care group, the mean age was higher (66 years) for those with incomplete data for the primary outcome measure than those with complete data (59 years). There were no other differences. Age was adjusted for in all analyses and included in the multiple imputation model, therefore this is unlikely to have unduly biased our results.

The economic evaluation included cost and utility data from baseline, 4 months and 24 months, and so there was greater scope for data to be missing. The proportion of participants with complete cost or utility data for all three time points (baseline, 4 months and 24 months) was lower than at the final time point (24 months) alone, suggesting that missing data items rather than loss to follow-up contributed to the level of missing data. Almost 70% of participants had complete cost *or* utility data; however, the proportion of participants with data for both was lower. As described in the Methods section, all available data were used to inform the imputed values and the role of multiple imputation can be thought of as filling in the blanks. A *post hoc* comparison of the characteristics of the participants with complete/incomplete economic data showed that only ethnicity was significantly different: there was a higher proportion of black and minority ethnic participants with complete data than incomplete data (see supplementary Table 2). This was surprising because for the participants who did not return the 24-month postal questionnaire, only English speakers were able to complete it over the telephone (because of limited resources). The cost-effectiveness results were somewhat sensitive to missing data. The direction of the effect on costs and QALYs were the same, but the cost to gain one QALY was higher in the complete case analysis.

Data on depression symptoms (SCL-D13), health status (EQ-5D-5L) and healthcare resource utilisation were self-reported by participants. Although pragmatic, self-reported methods are potentially prone to recall bias over a long-term follow-up. This is especially true in relation to capturing healthcare utilisation, particularly among those who use a large number of different healthcare services. However, use of costly services (e.g. an in-patient admission or major surgery) are notable events and likely to be recalled even over a long period.^30^^,^^31^ Furthermore, the intervention in this trial was not expected to affect recall of healthcare utilisation. Although total costs may be an underestimation, this is expected to be to the same extent in both groups and unlikely to influence the net cost. The second follow-up (at 24 months) had not been funded at the time of writing the original protocol for the COINCIDE trial, and so participants were only approached for second follow-up after the original study period had ended. If it had been possible to inform participants about the second follow-up earlier, the attrition rate may have been lower. However, a 68% retention rate over 24 months for the primary outcome measure is acceptable and, as described above, multiple imputation was used to minimise the effect of missing data. Additionally, we were only resourced to collect long-term follow-up data for the primary and economic outcomes, making it impossible to address questions about the long-term effect of the intervention on secondary outcomes evaluated in our short-term analysis.^9^

### Implications

A strong case for routinely using collaborative care as a framework of care for depression was made by the CADET trial.^27^^,^^32^ We have shown that collaborative care is as effective in people with long-term conditions as it is in people with depression alone.^7^ However, current NICE guidance about the management of multimorbidity has excluded evidence from interventions that primarily target depression. The rationale for this was that any benefits for physical health may be an indirect effect of improvements in mood. Although the COINCIDE trial was designed as a depression intervention, it has been identified as an approach that can effectively integrate the mental and physical healthcare of people with multimorbidity in primary care.^33^ In the COINCIDE trial, collaborative care improved both depression and physical functioning in people with multimorbidity. Furthermore, collaborative care is also likely to be cost-effective over the long term. These findings address an evidence gap identified by NICE when developing their guideline/recommendations regarding the management of multimorbidity.^8^

In conclusion, the need for cost-effective interventions for multimorbidity is of paramount importance in high-, middle-, and low-income regions faced with the burden of managing ageing and/or deprived populations with complex health needs. Despite the limitations of this analysis, it can be concluded that collaborative care is clinically effective (with a moderate effect size) and cost-effective over the long term, as a treatment for people with depression in the context of multimorbidity. In addition to existing evidence, findings from the COINCIDE trial send a strong signal to clinicians and commissioners in primary care that collaborative care merits implementation to manage the effect of mental–physical multimorbidity over the long term.

## Supplementary material

For supplementary material accompanying this paper visit https://doi.org/10.1192/bjp.2018.70. click here to view supplementary material

## References

[1] Dickens C, Katon W, Blakemore A, Does depression predict the use of urgent and unscheduled care by people with long term conditions? A systematic review with meta-analysis. J Psychosom Res 2012; 73: 334–42. 23062805 10.1016/j.jpsychores.2012.08.018

[2] Nunes BP, Flores TR, Mielke GI, Thumé E, Facchini LA. Multimorbidity and mortality in older adults: a systematic review and meta-analysis. Arch Gerontol Geriatr 2016; 67: 130–8. 27500661 10.1016/j.archger.2016.07.008

[3] Mujica-Mota RE, Roberts M, Abel G, Common patterns of morbidity and multi-morbidity and their impact on health-related quality of life: evidence from a national survey. Qual Life Res 2015; 24: 909–18. 25344816 10.1007/s11136-014-0820-7 PMC4366552

[4] Thorpe KE, Ogden LL, Galactionova K. Chronic conditions account for rise in Medicare spending from 1987 to 2006. Health Aff (Millwood) 2010; 29: 718–24. 20167626 10.1377/hlthaff.2009.0474

[5] Archer J, Bower P, Gilbody S, Collaborative care for depression and anxiety problems. Cochrane Database Syst Rev 2012; 10: CD006525. 10.1002/14651858.CD006525.pub2 PMC11627142 23076925

[6] Coventry PA, Lovell K, Dickens C, Collaborative Interventions for Circulation and Depression (COINCIDE): study protocol for a cluster randomized controlled trial of collaborative care for depression in people with diabetes and/or coronary heart disease. Trials 2012; 13: 139. 22906179 10.1186/1745-6215-13-139 PMC3519809

[7] Panagioti M, Bower P, Kontopantelis E, Association between chronic physical conditions and the effectiveness of collaborative care for depression. JAMA Psychiatry 2016; 73: 978. 27602561 10.1001/jamapsychiatry.2016.1794

[8] National Institute of Health and Care Excellence (NICE). *Multimorbidity: Clinical Assessment and Management [NG56]* NICE, 2016 (https://www.nice.org.uk/guidance/ng56).

[9] Coventry P, Lovell K, Dickens C, Integrated primary care for patients with mental and physical multimorbidity: cluster randomised controlled trial of collaborative care for patients with depression comorbid with diabetes or cardiovascular disease. BMJ 2015; 350: h638. 25687344 10.1136/bmj.h638 PMC4353275

[10] Coventry PA, Lovell K, Dickens C, Update on the collaborative interventions for circulation and depression (COINCIDE) trial: changes to planned methodology of a cluster randomized controlled trial of collaborative care for depression in people with diabetes and/or coronary heart disease. Trials 2013; 14: 136. 23663556 10.1186/1745-6215-14-136 PMC3660180

[11] Brown S, Thorpe H, Hawkins K, Brown J. Minimization – reducing predictability for multi-centre trials whilst retaining balance within centre. Stat Med 2005; 24: 3715–27. 16320287 10.1002/sim.2391

[12] Kroenke K, Spitzer RL, Williams JBW. The PHQ-9. J Gen Intern Med 2001; 16: 606–13. 11556941 10.1046/j.1525-1497.2001.016009606.x PMC1495268

[13] National Institute of Health and Care Excellence (NICE). *Depression in Adults With a Chronic Physical Health Problem: Recognition and Management [CG91]* NICE, 2009 (https://www.nice.org.uk/guidance/CG91). 39808015

[14] Derogatis LR, Cleary PA. Confirmation of the dimensional structure of the SCL-90: a study in construct validation. J Clin Psychol 1977; 33: 981–9.

[15] Herdman M, Gudex C, Lloyd A, Janssen MF, Kind P, Parkin D, Development and preliminary testing of the new five-level version of EQ-5D (EQ-5D-5L). Quality of life research 2011; 20(10): 1727–36. 21479777 10.1007/s11136-011-9903-x PMC3220807

[16] National Institute of Health and Care Excellence (NICE). *Guide to the Methods of Technology Appraisal.* NICE, 2013 (https://www.nice.org.uk/process/pmg9/chapter/foreword). 27905712

[17] Personal and Social Services Research Unit. *Unit Costs of Health and Social Care.* Personal and Social Services Research Unit, 2015 (https://www.pssru.ac.uk/project-pages/unit-costs/).

[18] Department of Health. *National Schedule of Reference Costs* Department of Health, 2015 (https://www.gov.uk/government/collections/nhs-reference-costs).

[19] Devlin N, Shah K, Feng Y, Mulhern B, Van Hout B. *Valuing Health-Related Quality of Life: An EQ-5D-5L Value Set for England* Office of Health Economics, 2016 (https://www.ohe.org/publications/valuing-health-related-quality-life-eq-5d-5l-value-set-england). 10.1002/hec.3564 PMC6680214 28833869

[20] van Hout B, Janssen MF, Feng Y-S, Interim scoring for the EQ-5D-5L: mapping the EQ-5D-5L to EQ-5D-3L value sets. Value Heal 2012; 15: 708–15. 10.1016/j.jval.2012.02.008 22867780

[21] Skevington SM, Lotfy M, O'Connell KA. The World Health Organization's WHOQOLBREF quality of life assessment: psychometric properties and results of the international field trial. A report from the WHOQOL group. Qual Life Res 2004; 13: 299–310. 15085902 10.1023/B:QURE.0000018486.91360.00

[22] Spitzer RL, Kroenke K, Williams JW, Löwe B. A brief measure for assessing generalized anxiety disorder: the GAD-7. Arch Intern Med 2006; 166: 1092–97. 16717171 10.1001/archinte.166.10.1092

[23] Lorig K, Stewart A, Ritter P, Gonzalez V, Laurent D, Lynch J. Outcome measures for health education and other health care interventions. Sage, 1996.

[24] Osborne RH, Elsworth GR, Whitfeld K. The health education impact questionnaire (heiQ): an outcomes and evaluation measure for patient education and self-management interventions for people with chronic conditions. Patient Educ Couns 2007; 66: 192–201. 17320338 10.1016/j.pec.2006.12.002

[25] Sheehan DV, Harnett-Sheehan K, Raj BA. The measurement of disability. Int Clin Psychopharmacol 1996; 11(suppl 3): 89–95. 10.1097/00004850-199606003-00015 8923116

[26] Gwirtsman H, Blehar M, McCullough J, Kocsis J, Prien R. Standardized assessment of dysthymia. Psychopharmacol Bull 1997; 33: 1–5. 9133745

[27] Green C, Richards DA, Hill JJ, Cost-effectiveness of collaborative care for depression in UK primary care: economic evaluation of a randomised controlled trial (CADET). PLoS One 2014; 9: e104225. 25121991 10.1371/journal.pone.0104225 PMC4133193

[28] Camacho EM, Ntais D, Coventry P, Long-term cost-effectiveness of collaborative care (*v.* usual care) for people with depression and comorbid diabetes or cardiovascular disease: a Markov model informed by the COINCIDE randomised controlled trial. BMJ Open 2016; 6: e012514. 10.1136/bmjopen-2016-012514 PMC5073527 27855101

[29] Barnett K, Mercer SW, Norbury M, Watt G, Wyke S, Guthrie B. Epidemiology of multimorbidity and implications for health care, research, and medical education: a cross-sectional study. Lancet 2012; 380: 37–43. 22579043 10.1016/S0140-6736(12)60240-2

[30] Petrou S, Murray L, Cooper P, Davidson LL. The accuracy of self-reported healthcare resource utilization in health economic studies. Int J Technol Assess Health Care 2002; 18: 705–10. 12391960 10.1017/s026646230200051x

[31] Bhandari A, Wagner T. Self-reported utilization of health care services: improving measurement and accuracy. Med Care Res Rev 2006; 63: 217–35. 16595412 10.1177/1077558705285298

[32] Richards DA, Hill JJ, Gask L, Clinical effectiveness of collaborative care for depression in UK primary care (CADET): cluster randomised controlled trial. BMJ 2013; 347: f4913. 23959152 10.1136/bmj.f4913 PMC3746956

[33] The King's Fund. *Long-Term Conditions and Multi-Morbidity* The King's Fund, 2015 (https://www.kingsfund.org.uk/time-to-think-differently/trends/disease-and-disability/long-term-conditions-multi-morbidity).

