# Supplementary material for: Long-term clinical and cost-effectiveness of collaborative care (versus usual care) for people with mental–physical multimorbidity: cluster-randomised trial
Source: Br J Psychiatry. 2018 Aug;213(2):456–63. doi: 10.1192/bjp.2018.70 (PMC6429252; doi:10.1192/bjp.2018.70)
Supplement: Supplementary file 1 [file S0007125018000703sup001.pdf]

Camacho et al - Supplementary material

It was possible to calculate utilities for the majority (69-95%) of participants at all time points and there was little difference in the proportion with complete and partial data. For costs however, it is clear that for a number of participants only partial data was reported, which is particularly notable at 4 months. At 24 months a similar proportion of participants had complete data for the different categories but at four months more participants had complete data for inpatient and emergency admissions than outpatient and primary care visits.

Overall, utility data were more complete than cost data. The proportion of participants with data for both costs and utility was somewhat lower than for either variable alone.

Table S1 Summary of available cost and utility data

	**N (%)** **[*% partial data*][a]**		
	**Cost**	**Utility**	**Cost *and* utility**
**Study time point**			
Baseline	n/a	366 (95) *[99]*	n/a
4 months	227 (59) *[84]*	316 (82) *[83]*	225 (58)
24 months	216 (56) *[68]*	266 (69) *[69]*	215 (56)
All	142 (37)	229 (59)	134 (35)

	**Participants with complete cost data by healthcare category** **N (%)**	
	4 months	24 months
Primary and community	276 (71)	248 (64)
Outpatient	269 (70)	260 (67)
Hospital day case	309 (80)	237 (61)
Inpatient	324 (84)	264 (68)
Emergency	323 (83)	256 (66)

[a]Response to at least one domain on EQ-5D and cost recorded for at least one category of resource use

Table S2 Baseline characteristics of participants with and without complete cost and utility data

	Complete cases (N=134)	Incomplete cases (N=253)	p-value for difference
Age, years (mean, SD)	58 (11.6)	59 (11.7)	0.37
Sex (% female)	42%	36%	0.26
Ethnicity (% non-white)	21%	10%<br>n=249	0.006*
Employment status (% in paid employment)	30%	23%	0.17
Number of conditions (mean, SD)	6 (3)	6 (3)	0.40
Baseline mean SCL score (mean, SD)	2.29 (0.77)	2.38 (0.76)	0.25
Baseline EQ-5D value (mean, SD)	0.562 (0.292)	0.517 (0.291)<br>N=232	0.16
Treatment allocation<br>(% collaborative care)	54%	49%	0.38

*statistically significant difference at 0.05 level

p-values derived using unadjusted logistic regression with completeness as a binary dependent variable

Table S3 Costs of healthcare resources used over 24 months and health state index, unadjusted values

	Collaborative care		Usual care	
	**N**	**Mean (SD)**	**N**	**Mean (SD)**
Primary & community care	85	£1610 (2094)	110	£1401 (2720)
Hospital outpatient visits	94	£1195 (2840)	108	£920 (1362)
Hospital day case visits	95	£738 (1823)	111	£788 (1906)
A & E visits	105	£163 (375)	127	£179 (303)
Hospital inpatient care	109	£3564 (9944)	133	£2697 (8925)
Intervention cost (including training)	£321 (168)		n/a	
Training cost	£130		n/a	
**All costs**	64	£8052 (14,398)	78	£4866 (8530)
Difference in total cost	3186			
(95% CI; p-value)	(-664, 7035; 0.104)			
**Health state (EQ-5D) index**	**N**	**Mean (SD)**	**N**	**Mean (SD)**
Baseline	181	0.548 (0.287)	185	0.519 (0.296)
4 months	152	0.603 (0.295)	164	0.557 (0.298)
24 months	121	0.609 (0.290)	145	0.500 (0.341)
**QALYs**	105	1.199 (0.553)	124	1.054 (0.585)
(between baseline and 24 months)				
Difference in QALYs	0.144			
(95% CI; p-value)	(-0.005, 0.294; 0.058)			

N= number of participants using a particular service; mean cost calculated for those using the respective service only (i.e. zeros are excluded)

Costs reported as GBP (£) standardised to single price year (2015-16)

QALY = quality adjusted life year

CONSORT checklist of information to include when reporting a randomised trial

Section/Topic	Item No	Checklist item	Reported on page No
**Title and abstract**			
	1a	Identification as a randomised trial in the title	1
	1b	Structured summary of trial design, methods, results, and conclusions (for specific guidance see CONSORT for abstracts)	3
**Introduction**			
Background and objectives	2a	Scientific background and explanation of rationale	4
	2b	Specific objectives or hypotheses	4
**Methods**			
Trial design	3a	Description of trial design (such as parallel, factorial) including allocation ratio	4
	3b	Important changes to methods after trial commencement (such as eligibility criteria), with reasons	n/a
Participants	4a	Eligibility criteria for participants	4
	4b	Settings and locations where the data were collected	4
Interventions	5	The interventions for each group with sufficient details to allow replication, including how and when they were actually administered	5
Outcomes	6a	Completely defined pre-specified primary and secondary outcome measures, including how and when they were assessed	5
	6b	Any changes to trial outcomes after the trial commenced, with reasons	n/a
Sample size	7a	How sample size was determined	4
	7b	When applicable, explanation of any interim analyses and stopping guidelines	n/a
Randomisation:			
Sequence generation	8a	Method used to generate the random allocation sequence	4
	8b	Type of randomisation; details of any restriction (such as	4

		blocking and block size)	
Allocation concealment mechanism	9	Mechanism used to implement the random allocation sequence (such as sequentially numbered containers), describing any steps taken to conceal the sequence until interventions were assigned	4
Implementation	10	Who generated the random allocation sequence, who enrolled participants, and who assigned participants to interventions	4
Blinding	11a	If done, who was blinded after assignment to interventions (for example, participants, care providers, those assessing outcomes) and how	n/a
	11b	If relevant, description of the similarity of interventions	n/a
Statistical methods	12a	Statistical methods used to compare groups for primary and secondary outcomes	6
	12b	Methods for additional analyses, such as subgroup analyses and adjusted analyses	6
**Results**			
Participant flow (a diagram is strongly recommended)	13a	For each group, the numbers of participants who were randomly assigned, received intended treatment, and were analysed for the primary outcome	7
	13b	For each group, losses and exclusions after randomisation, together with reasons	Fig S1
Recruitment	14a	Dates defining the periods of recruitment and follow-up	7
	14b	Why the trial ended or was stopped	as planned
Baseline data	15	A table showing baseline demographic and clinical characteristics for each group	Table 1
Numbers analysed	16	For each group, number of participants (denominator) included in each analysis and whether the analysis was by original assigned groups	All tables
Outcomes and estimation	17a	For each primary and secondary outcome, results for each group, and the estimated effect size and its precision (such as 95% confidence interval)	7-9; Table 2
	17b	For binary outcomes, presentation of both absolute and relative effect sizes is recommended	n/a
Ancillary analyses	18	Results of any other analyses performed, including subgroup analyses and adjusted analyses, distinguishing	

		pre-specified from exploratory	
Harms	19	All important harms or unintended effects in each group (for specific guidance see CONSORT for harms)	Table 2
**Discussion**			
Limitations	20	Trial limitations, addressing sources of potential bias, imprecision, and, if relevant, multiplicity of analyses	9-10
Generalisability	21	Generalisability (external validity, applicability) of the trial findings	9-10
Interpretation	22	Interpretation consistent with results, balancing benefits and harms, and considering other relevant evidence	11
**Other information**			
Registration	23	Registration number and name of trial registry	4
Protocol	24	Where the full trial protocol can be accessed, if available	4
Funding	25	Sources of funding and other support (such as supply of drugs), role of funders	11

CHEERS checklist—Items to include when reporting economic evaluations of health interventions

Section/item	Item No	Recommendation	Reported on
**Title and abstract**			
Title	1	Identify the study as an economic evaluation or use more specific terms such as "cost-effectiveness analysis", and describe the interventions compared.	Page 1
Abstract	2	Provide a structured summary of objectives, perspective, setting, methods (including study design and inputs), results (including base case and uncertainty analyses), and conclusions.	Page 3
**Introduction**			
Background and objectives	3	Provide an explicit statement of the broader context for the study.	Page 4
		Present the study question and its relevance for health policy or practice decisions.	Page 4
**Methods**			
Target population and subgroups	4	Describe characteristics of the base case population and subgroups analysed, including why they were chosen.	Page 5
Setting and location	5	State relevant aspects of the system(s) in which the decision(s) need(s) to be made.	Pages 4-5
Study perspective	6	Describe the perspective of the study and relate this to the costs being evaluated.	Page 6
Comparators	7	Describe the interventions or strategies being compared and state why they were chosen.	Page 5
Time horizon	8	State the time horizon(s) over which costs and consequences are being evaluated and say why appropriate.	Page 5
Discount rate	9	Report the choice of discount rate(s) used for costs and outcomes and say why appropriate.	Page 5
Choice of health outcomes	10	Describe what outcomes were used as the measure(s) of benefit in the evaluation and their relevance for the type of analysis performed.	Page 5
Measurement of effectiveness	11a	*Single study-based estimates:* Describe fully the design features of the single effectiveness study and why the single study was a sufficient source of clinical effectiveness data.	Pages 4-6
	11b	*Synthesis-based estimates:* Describe fully the methods used for identification of included studies and synthesis of clinical effectiveness data.	n/a
Measurement and valuation of preference based outcomes	12	If applicable, describe the population and methods used to elicit preferences for outcomes.	n/a
Estimating resources and costs	13	*Single study-based economic evaluation:* Describe approaches used to estimate resource use associated with the alternative interventions. Describe primary or secondary research methods for valuing each resource item in terms of its unit cost. Describe any adjustments made to approximate to opportunity costs.	Page 5

Section/item	Item No	Recommendation	Reported on
Currency, price date, and conversion	14	Report the dates of the estimated resource quantities and unit costs. Describe methods for adjusting estimated unit costs to the year reported costs if necessary. Describe methods for converting costs into a common currency base and the exchange rate.	Page 7 (dates) Page 6 (methods)
Choice of model	15	Describe and give reasons for the specific type of decision-analytical model used. Providing a figure to show model structure is strongly recommended.	n/a
Assumptions	16	Describe all structural or other assumptions underpinning the decision-analytical model.	n/a
Analytical methods	17	Describe all analytical methods supporting the evaluation. This could include methods for dealing with skewed, missing, or censored data; extrapolation methods; methods for pooling data; approaches to validate or make adjustments (such as half cycle corrections) to a model; and methods for handling population heterogeneity and uncertainty.	Pages 6-7
**Results**			
Study parameters	18	Report the values, ranges, references, and, if used, probability distributions for all parameters. Report reasons or sources for distributions used to represent uncertainty where appropriate. Providing a table to show the input values is strongly recommended.	n/a
Incremental costs and outcomes	19	For each intervention, report mean values for the main categories of estimated costs and outcomes of interest, as well as mean differences between the comparator groups. If applicable, report incremental cost-effectiveness ratios.	Page 16 Tables 3,S3
Characterising uncertainty	20	*Single study-based economic evaluation:* Describe the effects of sampling uncertainty for the estimated incremental cost and incremental effectiveness parameters, together with the impact of methodological assumptions (such as discount rate, study perspective).	Page 16 Table 3
Characterising heterogeneity	21	If applicable, report differences in costs, outcomes, or cost-effectiveness that can be explained by variations between subgroups of patients with different baseline characteristics or other observed variability in effects that are not reducible by more information.	n/a
**Discussion**			
Study findings, limitations, generalisability, and current knowledge	22	Summarise key study findings and describe how they support the conclusions reached. Discuss limitations and the generalisability of the findings and how the findings fit with current knowledge.	Pages 9-11
**Other**			
Source of funding	23	Describe how the study was funded and the role of the funder in the identification, design, conduct, and reporting of the analysis.	Page 11
Conflicts of interest	24	Describe any potential for conflict of interest of study contributors in accordance with journal policy.	Page 3
